# Supplementary figures and images for: Examining individual, health service, and experience of care determinants of patients’ satisfaction - A cross-sectional facility-based study in KP, Pakistan
Source: PLoS One. 2026 May 27;21(5):e0346788. doi: 10.1371/journal.pone.0346788 (PMC13215502; doi:10.1371/journal.pone.0346788)

Appendix

Appendix 1

Emojis associated to each modality of the satisfaction questions


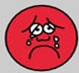

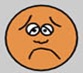

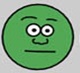

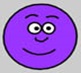

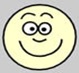

Supplement: S1 Appendix — (DOCX) [file pone.0346788.s001.docx]
