# Supplementary material for: Examining individual, health service, and experience of care determinants of patients’ satisfaction - A cross-sectional facility-based study in KP, Pakistan
Source: PLoS One. 2026 May 27;21(5):e0346788. doi: 10.1371/journal.pone.0346788 (PMC13215502; doi:10.1371/journal.pone.0346788)
Supplement: Attachment — Submitted filename: PATIENT SATISFACTION_ plosone_ReviewerComments.docx [file pone.0346788.s003.docx]

# What affects a patient’s satisfaction with healthcare? An empirical study from Patient Exit Interviews in Khyber Pakhtunkhwa, Pakistan

## Authors

Laurène PETITFOUR^a^, Stephan BRENNER^a^, Khalid REHMAN^b^, Swati Srivastava^a^

Asad ULLAH^b^, Zohaid KHAN^b^, Manuela De Allegri^a^

## Affiliations

a: Universitatsklinikum Heidelberg, Heidelberg Institute for Global Health (Heidelberg, Germany)

b: Office of Research, Innovation, and Commercialization (ORIC), (Peshawar, Khyber Pakhtunkhwa, Pakistan)

## Corresponding author

[Laurene.petitfour@ird.fr](mailto:Laurene.petitfour@ird.fr)

# Abstract

Health systems aim to meet populations’ needs and to spur their satisfaction, requiring to first understand what drives this satisfaction, whether these drivers come from the health system or not. In this empirical study, we use survey data from 666 patients exiting outpatient services of 60 public and 62 private health facilities in four districts of the province of Khyber Pakhtunkhwa, Pakistan.

We asked patients questions about their socio-economic situation and their satisfaction regarding several aspects of the healthcare they just received. We then investigated elements of this experience (respect for privacy, out-of-pocket expenditures, availability of drugs). First, we implemented a Principal Component Analysis to compute a composite Patient Satisfaction score. Second, we used this score as the outcome in a linear model to identify its determinants.

We found a generally high level of satisfaction regarding outpatient services within the sample, with an average satisfaction score of 76.37 out of 100. We did not find any association between satisfaction and the type of facility. Being a female patient and being insured by an inpatient-focused insurance were significantly associated with satisfaction (respectively positively and negatively). The variables that affected satisfaction the most were the characteristics of the healthcare experience, namely: privacy (positively) availability of drugs (positively) and cost of healthcare (negatively).

Our results highlight the importance of considering the specific needs of a subpopulation (e.g. female patients) to provide them with appropriate care and to ensure the respect of core aspects of healthcare experiences, in all types of facility.

# Keywords

Patient satisfaction; Khyber Pakhtunkhwa; primary healthcare facilities

# Introduction

## Background

Health system responsiveness is defined as “how well a health system meets the legitimate expectations of the people it serves” (1) (2) and is considered both as an objective *per se* and as an essential element in enabling Universal Health Coverage (UHC) (3). As such, the role that health system responsiveness plays is central to the quality health service provision and the establishment of trust and respect between providers and their patients (4,5). Responsive health services are more likely to ensure the recommended use of healthcare (6,7) and therefore more likely to result in better health outcomes (4), especially among chronically ill patients. Non-responsive healthcare provision, on the other hand, is more likely to contribute to poor treatment adherence among patients with chronic diseases (8).

Ideally, the assessment of health service responsiveness required information on how well the health system responds to legitimate patient expectations (9). In the absence of information to scrutinize whether expectations are legitimate or not, empirical studies commonly rely on the assessment of patient experience or satisfaction, acknowledging that some expectations might be illegitimate or not under the control of the service provider. The assessment of patient experiences with receiving health care commonly focuses on perceptions related to the physical health care environment (e.g., amenities, cleanliness), service organization (e.g., waiting time), the patient-provider interactions (e.g., technical and interpersonal quality, opportunity to ask questions), or the outcome and affordability of care (10).

In comparison, the assessment of patient satisfaction is primarily focused on how patients rate different healthcare experiences (11,12). Since patients’ satisfaction with health care experiences are likely to be influenced by factors not directly related to the service provider or the overall health system, understanding associations between measured satisfaction and contextual factors, such as socio-demographic determinants of health (e.g. age, gender, education, socio-economic status, marital status, race, religion), or access to and use of care (geographic characteristics, visit regularity, health status) is critical (13,14).

In Pakistan empirical evidence on patient satisfaction in the context of outpatient care is so far limited. Surveys of outpatients receiving care at a tertiary hospital of Karachi identified patient dissatisfaction with aspects of the patient-provider interaction, such as insufficient time to explain and answer questions and lack of privacy and respectfulness (15,16). Interviews with outpatients at a public diabetes clinic in Punjab province found positive patient satisfaction to be associated with competent provider communication, and was generally higher among patients who were female, uneducated, or employed (17). Studies conducted at hospitals and tertiary care centres found that both outpatients and inpatients were generally more satisfied with the care received from the private sector compared with public sector providers (18,19). While positive patient satisfaction with private providers was associated with prompt service delivery, satisfaction with private providers was associated with empathy for patients’ problems and needs (20).

Our study aims at identifying determinants of patients’ satisfaction with outpatient care services in the province of Khyber Pakhtunkhwa (KP), Pakistan. Our study adds to the existing literature by examining patients’ satisfaction in a remote region of the country, relying on a large sample of patients, drawn from a large sample of primary level facilities. It also uses a more comprehensive measure than what has been used in the country in the past, considering several aspects of the experience of care in the outpatient setting.

# Methods

## Study setting and sampling

Figure 1 Map of province Khyber Pakhtunkhwa, sample districts marked in blue

Our study area constituted four districts of Khyber Pakhtunkhwa province in Pakistan: Chitral, Kohat, Malakand and Mardan (see Figure 1). The healthcare delivery system in Khyber Pakhtunkhwa relies on both public and private facilities (see Figure 2).

Public sector healthcare delivery follows a pyramidal structure: the bottom tier consists of Basic Health Units (BHU), which commonly include two to five medical practitioners that provide primary outpatient care for a modest fee of 10 PKR. The second tier consists of Rural Health Centers (RHCs), which can vary in both their size and their range of primary and some specialty services, as well as inpatient care. The top tiers include Tehsil Head Quarter (THQs) and District Head Quarter (DHQs) hospitals, which offer both inpatient and outpatient care for a range of specialty services. The private sector consists of individual or group-based general practitioners (GP) or specialists providing facility- or office-based care. Group-based private providers usually consist of two or more practitioners offering services at facilities managed by a single private authority. In both private and public sector facilities direct user charges are the predominant modality, although these charges are highly subsidized in the latter.

**Figure 2 Healthcare system in Khyber Pakhtunkhwa**

## Sampling

We collected data from September to November 2022 and from March to April 2023 from a sample of 60 public and 62 private healthcare facilities providing primary level outpatient services. The public sample included a census of all DHQ and THQ hospitals, all RHCs, and 25 BHUs randomly selected from a total of 98 BHUs in the study area. The private sample included 62 facilities randomly selected from a list of 245 private facility provided to us by the KP Health Department. The 62 private facilities included 37 GPs (27 individual and 10 group-based practices), 11 clinical specialists (six individual, five group-based), and seven group-based outpatient service practices linked to an inpatient care facility.

Visiting each sampled provider, we conducted exit interviews with patients exiting the premises after having attended outpatient care services. Our teams were stationed with each provider for one day with the aim to reach eight patients per provider. We included only adult patients. Children or disabled patients that could not respond to the questionnaire on their own were excluded.

## Questionnaire

We collected information using a structured questionnaire, encompassing three sections. The first section collected information about a patient’s socio-demographic characteristics and household assets, which we used to estimate socio-economic status. The second section consisted of 21 survey questions collecting information on each patient’s satisfaction with the healthcare they had just received. The first four questions captured aspects of patients’ general satisfaction with the healthcare visit (e.g. whole care-seeking experience, likelihood to recommend the provider). The following 17 questions were related to specific aspects of the healthcare encounter (e.g., respect shown by provider, respect of confidentiality). A total of nine questions used a 5-item Likert scale to capture responses to questions phrased as: “How satisfied were you with <*SPECIFIC HEALTHCARE* *ASPECT>?”.* Scaled responses in the questionnaire were represented by five emoticons representing level of satisfaction ranging from “not smiling at all” to “much smiling” as depicted in Appendix 1, which was shown to the respondents to facilitate their understanding of the scale. The remaining eight questions were asked in a binary way (“Were you satisfied with <*SPECIFIC HEALTHCARE* *ASPECT>*?”), with responses recorded as “yes” or “no”. The set of four general satisfaction variables included 5-item Likert scale variables asking about the satisfaction regarding: care-seeking experience, rate of the quality of care, rate of the quality of care in relation to the price and likelihood to recommend this facility to others. The set of 17 variables for satisfaction regarding specific dimensions of the healthcare experience included nine 5-item Likert scale variables asking about the satisfaction regarding: respect shown by provider, confidentiality level, capacity to explain shown by provider, capacity to take time for questions shown by provider, capacity to take time shown by provider, waiting time, distance, adequacy of building, adequacy of staff. It further included eight binary satisfaction variables regarding: cleanliness, waiting room, smell, infrastructure cleanliness, access to clean toilet, access to clean water, access to soap, disability access. The third section focused on aspects of patients’ experience with the care received, which included questions about the availability of medication at the facility and the occurrence and amount of any out-of-pocket expenditures (OOPE) for medical and non-medical items.

## Data analysis

Data analysis followed two stages. First, we derived a measure of patient satisfaction using information from the second questionnaire section. Next, we used this patient satisfaction metric as a dependent variable in a multivariate model examining the relation between satisfaction and the individual’s socio-demographic profile and experience of care. The two stages are detailed below. We used Stata 15 to perform analyses.

### Construction of the composite satisfaction Index

During the first stage, we synthesized the information captured by all 21 questions related to satisfaction into one measure. Following the literature, we considered only aspects of satisfaction at this stage and did not yet consider actual elements of the healthcare experience (21). We then computed the correlation matrix for these 21 satisfaction variables to check that they were all significantly and positively correlated. Next, we conducted a Principal Component Analysis (PCA) using only the 17 variables measuring satisfaction with specific dimensions of the healthcare experience (i.e., we excluded the four variables on general satisfaction). Six variables were then removed because of missing values or limited explanatory power for the first principal component. Ultimately, the final PCA model included 11 of the initial 21 variables (see Table 1). In this model, the first component explained 41% of the total variability. We normalized eigenvalues to range from 0 to 100 to ease interpretation. Hereafter, we refer to the derived composite variable as the Satisfaction Score.

**Table 1: Variable content included in the Satisfaction Score**

| Eigenvalue of Component 1= 4.99 | Proportion of variability explained by the first component | 41% |
| --- | --- | --- |
|  | Variables | Coef |
| How satisfied were you with the… | Respect shown by provider? | 0.34 |
|  | Confidentiality level? | 0.30 |
|  | Capacity to explain shown by provider? | 0.36 |
|  | Capacity to take time for questions shown by provider? | 0.34 |
|  | Capacity to take time shown by provider? | 0.38 |
|  | Waiting time? | 0.30 |
|  | Distance? | 0.26 |
|  | Adequacy of building? | 0.28 |
|  | Adequacy of staff? | 0.33 |
| Were you satisfied with the | Cleanliness? | 0.13 |
|  | Waiting room? | 0.13 |
|  | Smell? | 0.15 |

### Multivariate analysis of satisfaction score

During the second stage, we identified factors associated with patient satisfaction related to service delivery using a linear estimator to explain the composite score of satisfaction. We identified and selected exposure variables based on existing literature (13,22). We introduced three types of exposure variables: variables related to the healthcare environment, variables related to patient characteristics, and variables related to a patient’s experience with receiving care. All variables and their measurement are listed in Table 2. Our final model specification was the following:

$$Y_{i,j,k}= \alpha+\beta_{k}D+\gamma_{j}F+\delta_{i}X+\varepsilon_{i}$$

Where Y represents the Satisfaction Score of individual i in district k and facility j, $\alpha$ represents a constant, D represents a matrix of binary variables taking the value of 1 when an individual was surveyed in district k and 0 otherwise, F represents a matrix of binary variables taking the value of 1 when the individual was surveyed in a facility category j and 0 otherwise, and X represents a matrix of variables pertaining to characteristics and experiences of seeking healthcare of individual i. Finally, $\varepsilon_{i}$ represents the standard error term. We clustered the standard errors at the facility level, as the number of interviewed patients per facility varied.

**Table 2: Exposure variables of the multivariate model**

| **Exposure variables** | **Type of variables: modalities** | **Expected sign and associated literature** |
| --- | --- | --- |
| **Environment** | | |
| District | 1 - Chitral  2 - Mardan  3 - Kohat/  4 - Malakand | Unknown |
| Type of facility | 1 - BHU  2 - RHC  3 - Hospitals  4 - Single practitioner private facility  5 - Group-based private facility | Higher satisfaction in private facilities (18,19) |
| **Individual characteristics** | | |
| Asset quintile | 1 - Lowest Asset Index quintile  2 - Second Lowest Asset Index quintile  3 - Middle Asset Index quintile  4 - Second Highest Asset Index quintile  5 - Highest Asset Index quintile | Higher satisfaction in private facilities (13,22) |
| Age | 1 - 18/25-year-old  2 - 26/39-year-old  3 - 40/54-year-old  4 - More than 55-year-old | Higher satisfaction with older age (13,22) |
| Gender | 0 - Male  1 - Female | Higher satisfaction for female (13,22) |
| BISP* beneficiary | 0 - No  1 - Yes |  |
| Education | 1 - Completed Primary  2 - Completed Secondary, Completed university degree, Postgraduate  3 - No Formal Schooling or University Degree  4 - Other: Religious education (Madrasa), certificate | Higher satisfaction for better educated individuals (13,22) |
| Sehat Plus card owner | Categorical: Yes/No | Higher satisfaction for insured individuals |
| **Characteristics of the experience with healthcare seeking** | | |
| Was privacy maintained throughout your care? | 0 - No  1 - Yes | Higher satisfaction when privacy is respected (16,23) |
| Did you receive all the drugs you were prescribed? | 0 - No  1 - Yes | Higher satisfaction when drugs are available (24) |
| Amount of OOPE | Continuous (ln transformed) | Lower satisfaction when OOPE are high (25) |
|  | 1 - No OOPE  2 - Between 1 and 10 PKR  3 - Between 11 and 800 PKR  4 - More than 800 PKR |  |

* BISP = Benazir Income Support Programme

Variables were introduced to the model following the empirical literature. The asset index was generated using Multiple Correspondence Analysis (MCA), building on the ownership of assets in the patient’s household to estimate the socio-economic status (SES) of the patient. The MCA included the ownership of refrigerator, washing machine, sofa, chair, cabinet, internet connection, bank account, the composition of the roof and walls. For ease of interpretation, we categorized people into SES quintiles. We included a binary variable indicating whether a patient was covered by the *Sehat Plus Card*, an inpatient health insurance program, and a binary variable indicating whether a patient was a beneficiary of the *Benazir Income Support Programme* (BISP), a wide-scale cash transfer social protection program. The rationale was to test the hypothesis that benefitting from a social protection program might increase familiarity and satisfaction with the local health system.

In addition, we also included three aspects of patient experience related to receiving healthcare previously used in the literature and considered relevant by the field team to identify areas of improvement with respect to patient satisfaction. This included privacy (16,23), drug availability (24), and the payment of out-of-pocket expenditures (25). In our study setting, patient privacy was not always guaranteed due to inappropriate infrastructures or provider oversight, making patients potentially uncomfortable and dissatisfied. The unavailability of drugs has been a major challenge in primary healthcare facilities, both public and private, requiring patients to buy prescribed drugs from private pharmacies, thus incurring additional out-of-pocket expenditures. We also included the amount of medical out-of-pocket expenditures (OOPE) in logarithmic form to control for extreme values. We further tested potential non-linearity in the effect of OOPE using several categorical dummy variables: no OOPE, between 1 and 10 PKR, between 11 and 800 PKR and more than 800 PKR (all compared to the reference category of 10 PKR). Thresholds were chosen purposively to isolate patients who did not incur any OOPE, to distinguish between patients who paid the 10 PKR forfeit from patients paying higher OOPE, and 800 PKR to isolate the highest quintile of OOPE. We tested our model for both the logarithmic of OOPE and its square.

## Ethics

This project received the approval of the Ethic committee of the Medical Faculty of the University of Heidelberg (S-492/2022) and received the ethics approval from Khyber Medical University ethics committee (KMU/EB/SI/00097). Written informed consent was obtained from each participant, and researchers had no access to information that could identify individual participants during or after data collection

# Results

Our sample consisted of 666 consenting respondents, of which 71% exited outpatient services of public facilities. The team could not reach the target of eight patients for all providers due to a low patient influx and not due to a refusal of consent. The respondent sample was quite balanced with regard to gender. Respondents with no education represented almost half of the sample (Table 3). Beneficiaries of the BISP social program represented nearly one quarter of the sample, and almost one half of them (45%) were enrolled in the inpatient healthcare insurance (Sehat Plus Card). With respect to patient experiences with receiving healthcare, 79% of respondents reported that their privacy was protected, while only one half was able to receive all prescribed medicines directly from the provider. OOPE were unevenly distributed, with 12% of respondents encountering no OOPE and 45% paying a 10 PKR forfeit for their healthcare. The remaining respondents incurred OOPE ranging between 11 and 14900 PKR, with a higher density in lower values. Only 22% of respondents paid more than 800 PKR for their healthcare (Table 3). Patient satisfaction was overall high, with an average and median score of 76 out of 100.

## Bivariate statistics

Table 3 presents the mean satisfaction score for each exposure variable including the associated standard error and F-statistic corresponding to the equality of means across modalities tested. For all but two variables (i.e., facility type and age), we rejected the null hypothesis of equal means across modalities. The variables with the largest difference in mean satisfaction across modalities included gender, the Sehat Plus Card, and all variables related to the experience with healthcare.

**Table 3: Distribution of exposure variables and bivariate statistics**

| **Global statistics on satisfaction** | | | | | |
| --- | --- | --- | --- | --- | --- |
| Satisfaction score | No. | Mean | Std. deviation. | Median |  |
| Total | 666 | 76.37 | 19.61 | 77.36 |  |
| **Environmental variables** | | | | | |
| District | No. | % | Mean Satisfaction score | Stand. err. Of Satisfaction score | F stat (pvalue) |
| Chitral | 93 | 14 | 72.07 | 2.12 | **7.57(0.0001)** |
| Mardan | 270 | 40.5 | 75.27 | 1.26 |  |
| Kohat | 148 | 22.2 | 74.59 | 1.61 |  |
| Malakand | 155 | 23.3 | 82.58 | 1.26 |  |
| Type of facility | No. | % | Mean Satisfaction score | Stand. err. Of Satisfaction score | F stat (pvalue) |
| BHU | 100 | 15 | 74.81 | 2.01 | 1.77(0.1337) |
| RHC | 183 | 27.5 | 77.66 | 1.51 |  |
| Hospitals | 192 | 28.8 | 74.28 | 1.38 |  |
| Single practitioner private facility | 105 | 15.8 | 79.89 | 2.02 |  |
| Group-based private facility | 86 | 12.9 | 75.84 | 1.75 |  |
| **Individual variables** | | | | | |
| Asset quintile | No. | % | Mean Satisfaction score | Stand. err. Of Satisfaction score | F stat (pvalue) |
| 1 | 119 | 17.9 | 68.66 | 1.83 | **24.33(<0.001)** |
| 2 | 141 | 21.2 | 72.34 | 1.54 |  |
| 3 | 137 | 20.6 | 79.91 | 1.72 |  |
| 4 | 122 | 18.3 | 77.99 | 1.87 |  |
| 5 | 147 | 22.1 | 81.86 | 1.36 |  |
| Age | No. | % | Mean Satisfaction score | Stand. err. Of Satisfaction score | F stat (pvalue) |
| 18/25-year-old | 149 | 22.4 | 73.71 | 1.65 | 1.88(0.1309) |
| 26/39-year-old | 268 | 40.2 | 77.99 | 1.20 |  |
| 40/54-year-old | 163 | 24.5 | 75.31 | 1.51 |  |
| More than 55-year-old | 86 | 12.9 | 77.97 | 2.03 |  |
| Total | 666 | 100 | 76.09 | 74.62 |  |
| Gender | No. | % | Mean Satisfaction score | Stand. err. Of Satisfaction score | F stat (pvalue) |
| Male | 340 | 51.1 | 85.83 | 0.85 | **213.18(<0.001)** |
| Female | 326 | 48.9 | 66.51 | 1.02 |  |
| BISP beneficiary? | No. | % | Mean Satisfaction score | Stand. err. Of Satisfaction score | F stat (pvalue) |
| No | 509 | 76.4 | 75.55 | 0.86 | **3.88(0.0494)** |
| Yes | 157 | 23.6 | 79.06 | 1.63 |  |
| Sehat Plus card | No. | % | Mean Satisfaction score | Stand. err. Of Satisfaction score | F stat (pvalue) |
| No | 360 | 54.1 | 71.06 | 0.99 | **62.93(<0.001)** |
| Yes | 306 | 45.9 | 82.63 | 1.07 |  |
| Education | No. | % | Mean Satisfaction score | Stand. err. Of Satisfaction score | F stat (pvalue) |
| Completed Primary | 137 | 20.6 | 82.69 | 1.60 | **11.03(<0.001)** |
| Completed Secondary /university degree | 193 | 29 | 77.99 | 1.29 |  |
| No Formal Schooling | 287 | 43.1 | 71.84 | 1.18 |  |
| Other: certificate, madrasa | 49 | 7.4 | 78.91 | 2.83 |  |
| **Aspects of the experience with seeking healthcare** | | | | | |
| Was privacy maintained throughout your care? | No. | % | Mean Satisfaction score | Stand. err. Of Satisfaction score | F stat (pvalue) |
| No | 139 | 20.9 | 69.71 | 2.01 | **20.89(<0.001)** |
| Yes | 527 | 79.1 | 78.13 | 0.78 |  |
| Dis you receive all the drugs you were prescribed? | No. | % | Mean Satisfaction score | Stand. err. Of Satisfaction score | F stat (pvalue) |
| No | 339 | 50.9 | 72.76 | 1.05 | **24.33(<0.001)** |
| Yes | 327 | 49.1 | 80.13 | 1.06 |  |
| Amount of OOPE | No. | % | Mean Satisfaction score | Stand. err. Of Satisfaction score | F stat (pvalue) |
| No OOPE | 77 | 11.6 | 64.95 | 2.02 | **12.92(<0.001)** |
| Between 1 and 10 PKR | 310 | 46.7 | 79.54 | 1.10 |  |
| Between 11 and 800 PKR | 130 | 19.6 | 74.04 | 1.71 |  |
| More than 800 PKR | 147 | 22.1 | 77.75 | 1.56 |  |
|  |  |  |  |  |  |
| Satisfaction score | No. |  | Mean | Std. deviation. | Median |
| Total | 666 |  | 76.37 | 19.61 | 77.36 |

## Multivariate analysis

Table 4 presents the results of the multivariate analysis with OOPE in logarithmic form (column a) and as categorical dummies (column b). Both models explained about 40% of patient satisfaction variability.

Among an individual’s characteristics, gender and inpatient health insurance coverage displayed the strongest association with satisfaction. Being female decreased the satisfaction score by 16 points (p-value <0.01), while having a Sehat Plus Card increased the satisfaction score by about 6 points (p-value <0.01). The remaining individual characteristics did not substantially affect satisfaction, and neither did the type of facility.

Variables related to the experience of care had significant effect on patient satisfaction. Specifically, privacy protection during the care encounter increased the satisfaction score by 12 points (p-value <0.01), and receiving all prescribed drugs was associated with a significant increase by 5 points (p-value <0.01).

The association between OOPE and patient satisfaction exhibited a nonlinear relationship. In column (a) both the logarithm and the squared logarithm of the OOPE amount was significantly associated yet displayed contrary directions. The amount of OOPE had a positive and then a negative effect on patient satisfaction (reversed U-curve). The turning point between the linear and the quadratic term was at 18.46 PKR and was consistent with the results presented in column (b). Compared with the reference group that paid 10 PKR, both patients who did not incur any OOPE (-8 percentage points, p-value<0.01) and those who paid between 11 and 800 PKR (-4 percentage points, p-value=0.05) had lower satisfaction scores.

**Table 4: Determinants of the Satisfaction Index (linear model estimations)**

|  | **VARIABLES** | **Outcome: Satisfaction score (column a)**  **Score (std. error)** | **Outcome: Satisfaction score (column b)**  **Score (std. error)** |
| --- | --- | --- | --- |
| **Environment** | **Districts (reference: Mardan)** |  |  |
|  | Chitral | -4.519 (3.114) | -20.6316 |
|  | Kohat | -0.966 (2.311) | -1.995 (2.418) |
|  | Malakand | 4.010* (2.384) | 3.575 (2.349) |
|  | **Types of facilities (reference: BHU)** |  |  |
|  | RHCs | 0.213 (3.095) | 0.277 (3.017) |
|  | DHQ and THQ Hospitals | -4.056 (2.585) | -3.548 (2.598) |
|  | Private single practitioner | -4.408 (3.775) | -5.326 (3.943) |
|  | Private group-based facilities | -3.390 (4.037) | -6.083 (3.809) |
| **Individual characteristics** | **Gender (reference: male)** |  |  |
|  | Female | -16.37*** (2.139) | -16.59*** (2.224) |
|  | **Age (reference: 26/39-year-old)** |  |  |
|  | 18/25-year-old | -5.51723 | -2.860 (1.731) |
|  | 40/54-year-old | -3.65284 | -3.42998 |
|  | More than 55-year-old | -1.300 (2.237) | -1.077 (2.240) |
|  | **Education (reference: no formal schooling)** |  |  |
|  | Completed Primary | 2.090 (1.787) | 1.641 (1.819) |
|  | Completed Secondary/University degree | -0.565 (1.740) | -1.213 (1.778) |
|  | Diploma/certif. | 2.092 (2.620) | 1.599 (2.656) |
|  | **Asset Index (reference: poorest quintile)** |  |  |
|  | Asset Index quintile 2 | 2.095 (2.125) | 1.967 (2.125) |
|  | Asset Index quintile 3 | 4.174* (2.517) | 3.868 (2.548) |
|  | Asset Index quintile 4 | 2.492 (2.800) | 2.417 (2.759) |
|  | Asset Index quintile 5 | 4.308* (2.599) | 4.206 (2.587) |
|  | **Sehat Plus card beneficiary (reference: No)** | 6.652*** (1.769) | 6.216*** (1.808) |
|  | **BISP BEN beneficiary (reference: No)** | 1.710 (1.899) | 1.632 (1.905) |
| **Experience with receiving healthcare** | **Privacy** | 11.82*** (3.020) | 11.92*** (3.063) |
|  | **Received all prescribed drugs** | 5.028*** (1.819) | 4.778** (1.847) |
|  | **Out-of-pocket expenditures (reference:10 PKR)** |  |  |
|  | No OOPE |  | -8.128*** (2.473) |
|  | Between 11 and 800 PKR |  | -4.180* (2.130) |
|  | Above 800 PKR |  | -3.716 (2.779) |
|  | Ln (med expenses) | 4.016*** (1.271) |  |
|  | Ln (med expenses) squared | -0.523*** (0.167) |  |
|  | Constant | 65.31*** | 73.79*** |
|  | Observations | 664 | 666 |
|  | R-squared | 0.412 | 0.407 |
|  | Robust standard errors in parentheses, *** p<0.01, ** p<0.05, * p<0.1 | | |

# Discussion

Relying on original survey data from 666 patients exiting the outpatient services of public and private providers, this study assessed the satisfaction of patients in relation to their experience with receiving care and examined factors associated with high satisfaction. Our findings indicate that satisfaction was generally high, but significantly lower for women. We found higher satisfaction rates associated with beneficiaries of the inpatient health insurance scheme available in the study area, with patients whose privacy has been protected during the care encounter, and with patients who received all prescribed drugs. We also found that patients who paid small amounts of OOPE were more satisfied compared with both those not paying OOPE at all and with those paying greater amounts.

The high level of satisfaction detected is in line with the prior literature on patient satisfaction in Pakistan. Previous estimations of satisfaction were 64% in a sample of 380 geriatric patients in one tertiary public hospital in Karachi (26) and 87% (very satisfied and satisfied) in a sample of 1,095 diabetes patients exiting a public diabetes clinic in Lahore (17). One study observed an increase in the proportion of satisfied patients, exiting outpatient and gynaecological services, from 34% to 82% over the one-year period following the implementation of an intervention consisting of quality of care workshops sensitizing and training staff in more patient-centred interactions with women (27). Other studies from Pakistan did not indicate the proportion of satisfied patients, but instead focused on relationships between patient satisfaction and patient expectation with the provider interaction (15,16,18,20).

This variability in findings is due to several reasons. First, the patient samples used across studies are not fully comparable in size and composition with respect to the care providers (facilities of different or similar types) or the type of care studied (general, diabetes, geriatric). Sample and care-specific differences can explain gaps in gender, age and socio-economic characteristics that likely affect a patient’s satisfaction with healthcare. Second, measures used to estimate patient satisfaction are diverse in their content and definition. Studies using the SERVQUAL questionnaire, for instance, do not usually compute a synthetic measure of satisfaction but rather derive and compare satisfaction levels for each of five domains (reliability, responsiveness, assurance, empathy and tangibility) (20,27,28). Other studies only assess patient’s satisfaction with a more general level of care (26) or derive satisfaction measures based on interpersonal care aspects only (28).

By comparing patients across various provider types using several aspects of the healthcare experience, we were able to provide a more comprehensive assessment of patient satisfaction, which contributes to and expands the currently available literature. Contrary to previous studies, we did not find that patients receiving care from private providers were more satisfied compared with public providers (18,19). This might be explained by the inclusion of additional respondent characteristics and healthcare experiences in our model, which had a stronger effect on patient satisfaction compared with the type of facility alone.

The effect of gender on patient satisfaction has been contradictory in the literature. For instance, a review of determinants of patient satisfaction included seven studies that identified higher satisfaction levels among males and six studies that identified the contrary (13). The relationship between patient gender and satisfaction is likely embedded in the local context and study setting and will require further investigations. For KP, the lower satisfaction of female patients might have been a reflection on the lack of female providers, both in public and private care settings, restricting provider choice for those women with a preference for female providers (29) and not corresponding to women’s specific expectations.

While the positive impact of health insurance coverage on patient satisfaction has been observed previously (30), the significant and positive impact of the Sehat Plus inpatient scheme might seem surprising because patients did not benefit from any of its advantages in outpatient services. While we cannot explain this association with certainty, it might be plausible that members of a social protection program are more familiar with or exposed to the healthcare system and value experiences in outpatient settings more favourably. More research, including qualitative studies, is needed to further understand the role of social protection schemes on patient experiences and satisfaction levels.

In our study, healthcare seeking experiences are crucial factors of patient satisfaction. For instance, privacy protection during the care process has been positively associated with patient satisfaction. Indeed, patients who reported their privacy or confidentiality invaded, were more likely to refuse additional examinations or less likely to retain information shared by their provider, thus negatively impacting diagnostic accuracy or treatment efficacy (16). Similarly, the availability of drugs was a major factor in determining positive patient satisfaction in our model, as drug shortages are a common occurrence in Pakistani healthcare facilities, and regularly cause treatment delays (31).

The reverse U-shape relationship between OOPE and satisfaction with a rather low turning point (18 PKR) confirms previous results on the importance of affordability in the assessment of the healthcare experience by patients (25,32). Patients seem not to sanction low levels of OOPE in terms of satisfaction but do so for amounts higher than 18 PKR. Given the negative impact of OOPE in terms of healthcare access and inequity reduction, the use and amount of user charges requires diligent attention and control to not contribute to the impoverishment or non-use of healthcare.

We acknowledge limitations in our study. First, some dimensions of patient experience were not captured by our questionnaire. For instance, we did not assess the reasons why patients sought care, such as the type or severity of their health condition. Hence, we might have omitted additional parameters in more comprehensively determining patients’ expectations and experiences of healthcare that might have differently determined their satisfaction. Second, more precise information about the characteristics of the providers (their training, their personal attitudes, their motivation) with whom the patient interacted would enrich the analysis. Third, the size of our sample, and the precise geographic setting might limit the overall generalizability of our findings to other settings. Fourth, the focus of our study is on outpatient care users, i.e., people who chose to seek care from the studied providers, which might have biased our satisfaction outcome to higher levels when compared with the population as a whole, since people with prior negative experiences of care might have been less likely to use the services again. Lastly, our study bears the inherent limitations of patient satisfaction measures, meaning that it does not account for the legitimacy of a patient’s expectations.

# Conclusion

Thanks to a diverse study sample, our empirical study offers a valuable contribution to the literature on the determinants of satisfaction and some actionable priorities to make the health system in KP more responsive to population needs and expectations. Satisfaction of patients exiting outpatient services is generally high, but with some discrepancies that allow us to identify areas to work on. First and foremost, respect for privacy, affordability and availability of appropriate treatment are essential to a positive experience with the healthcare system and should be monitored closely in all types of facilities. Additionally, the interpretation of the association between gender and satisfaction would require a deeper understanding of a patient’s expectations and needs regarding the healthcare system, including per subpopulation (e.g. gender and age), mobilizing both researchers and policymakers. This additional layer would allow to make more informed decisions in terms of policy choices needed to build a more responsive system.

# Acknowledgements

We gratefully acknowledge financial support as part of a scientific collaboration with the KfW Development Bank (“Scientific implementation research on outpatient-department (OPD) services in the social health protection initiative, Khyber Pakhtunkhwa (KP) province and the Gilgit Baltistan (GB) area”).

# Funding

Open Access funding enabled and organized by Projekt DEAL. We gratefully acknowledge financial support as part of a scientific collaboration with the KfW Development Bank (“Scientific implementation research on outpatient-department (OPD) services in the social health protection initiative, Khyber Pakhtunkhwa (KP) province and the Gilgit Baltistan (GB) area”).

# References

1.	Darby C, Valentine N, De Silva A, Murray CJL, Policy WHOGP on E for H. World Health Organization (WHO) : strategy on measuring responsiveness. 2003 [cited 2024 Apr 25]; Available from: https://iris.who.int/handle/10665/68703

2.	de Silva A. A Framework for Measuring Responsiveness. Geneva: World Health Organization; 2000. Report No.: 32.

3.	WHO. The World Health Report 2000: Health Systems : Improving Performance. World Health Organization; 2000. 244 p.

4.	Mirzoev T, Kane S. What is health systems responsiveness? Review of existing knowledge and proposed conceptual framework. BMJ Glob Health. 2017 Oct 31;2(4):e000486.

5.	Hanefeld J, Powell-Jackson T, Balabanova D. Understanding and measuring quality of care: dealing with complexity. Bull World Health Organ. 2017 May 1;95(5):368–74.

6.	Ratanawongsa N, Karter AJ, Parker MM, Lyles CR, Heisler M, Moffet HH, et al. Communication and medication refill adherence: the Diabetes Study of Northern California. JAMA Intern Med. 2013 Feb 11;173(3):210–8.

7.	Zolnierek KBH, Dimatteo MR. Physician communication and patient adherence to treatment: a meta-analysis. Med Care. 2009 Aug;47(8):826–34.

8.	Allotey P, Davey T, Reidpath DD. NCDs in low and middle-income countries - assessing the capacity of health systems to respond to population needs. BMC Public Health. 2014 Jun 20;14(Suppl 2):S1.

9.	Busse R. Chapter 9: Citizen and Patient Satisfaction, Responsive- ness and Experience with the Health System – Role and Contribution to Performance Assessment. In: Health System Performance Comparison. 2013. p. 255–79.

10.	Jenkinson C. The Picker Patient Experience Questionnaire: development and validation using data from in-patient surveys in five countries. International Journal for Quality in Health Care. 2002 Oct 1;14(5):353–8.

11.	Geldsetzer P, Haakenstad A, James EK, Atun R. Non-technical health care quality and health system responsiveness in middle-income countries: a cross-sectional study in China, Ghana, India, Mexico, Russia, and South Africa. J Glob Health. 2018;8(2):020417.

12.	Ratcliffe HL, Bell G, Awoonor-Williams K, Bitton A, Kim JH, Lipstiz S, et al. Towards patient-centred care in Ghana: health system responsiveness, self-rated health and experiential quality in a nationally representative survey. BMJ Open Qual. 2020 May;9(2):e000886.

13.	Batbaatar E, Dorjdagva J, Luvsannyam A, Savino MM, Amenta P. Determinants of patient satisfaction: a systematic review. Perspect Public Health. 2017 Mar;137(2):89–101.

14.	Srivastava A, Avan BI, Rajbangshi P, Bhattacharyya S. Determinants of women’s satisfaction with maternal health care: a review of literature from developing countries. BMC Pregnancy Childbirth. 2015 Dec;15(1):97.

15.	Imam SZ, Syed KS, Ali SA, Ali SU, Fatima K, Gill M, et al. Patients’ satisfaction and opinions of their experiences during admission in a tertiary care hospital in Pakistan – a cross sectional study. BMC Health Serv Res. 2007 Dec;7(1):161.

16.	Saleem SG, Ali S, Ghouri N, Maroof Q, Jamal MI, Aziz T, et al. Patient perception regarding privacy and confidentiality: A study from the emergency department of a tertiary care hospital in Karachi, Pakistan. Pak J Med Sci. 2022 Jan;38(2):351–5.

17.	Jalil A, Zakar R, Zakar MZ, Fischer F. Patient satisfaction with doctor-patient interactions: a mixed methods study among diabetes mellitus patients in Pakistan. BMC Health Serv Res. 2017 Feb 21;17(1):155.

18.	Shabbir A, Malik SA. Measuring patients’ healthcare service quality perceptions, satisfaction, and loyalty in public and private sector hospitals in Pakistan. van der Wiele T, editor. International Journal of Quality & Reliability Management [Internet]. 2016 Jan 1 [cited 2024 May 3];33(5). Available from: https://doi.org/10.1108/IJQRM-06-2014-0074

19.	Ghazanfar A, Idress IN, Zia Z, Munir N, Maryam S. Comparison of patients satisfaction levels in public and private tertiary care centres. J Pak Med Assoc. 2017 Aug;67(8):1305–8.

20.	Javed SA, Liu S, Mahmoudi A, Nawaz M. Patients’ satisfaction and public and private sectors’ health care service quality in Pakistan: Application of grey decision analysis approaches. Int J Health Plann Manage. 2019 Jan;34(1):e168–82.

21.	Larson E, Sharma J, Bohren MA, Tunçalp Ö. When the patient is the expert: measuring patient experience and satisfaction with care. Bull World Health Organ. 2019 Aug 1;97(8):563–9.

22.	Akthar N, Nayak S, Pai P Y. Determinants of patient satisfaction in Asia: Evidence from systematic review of literature. Clinical Epidemiology and Global Health. 2023 Sep;23:101393.

23.	Pratiwi AB, Padmawati RS, Willems DL. Behind open doors: Patient privacy and the impact of design in primary health care, a qualitative study in Indonesia. Front Med (Lausanne). 2022 Oct 19;9:915237.

24.	Khamis K, Njau B. Patients’ level of satisfaction on quality of health care at Mwananyamala hospital in Dar es Salaam, Tanzania. BMC Health Serv Res. 2014 Dec;14(1):400.

25.	Liang H, Xue Y, Zhang Z ruo. Patient satisfaction in China: a national survey of inpatients and outpatients. BMJ Open. 2021 Sep;11(9):e049570.

26.	Saleem T, Khalid U, Qidwai W. Geriatric patients’ expectations of their physicians: findings from a tertiary care hospital in Pakistan. BMC Health Services Research. 2009 Nov 13;9(1):205.

27.	Shaikh, Mobeen, Azam, Rabbani. Using SERVQUAL for assessing and improving patient satisfaction at a rural health facility in Pakistan. Eastern Mediterranean Health Journal. 2008;14(2):447–56.

28.	Javed SA, Ilyas F. Service quality and satisfaction in healthcare sector of Pakistan- the patients’ expectations. Int J Health Care Qual Assur. 2018 Jul 9;31(6):489–501.

29.	Shaukat M, Imping A, Rogge L, Khalid F, Ullah S, Ahmad F, et al. Un/met: a mixed-methods study on primary healthcare needs of the poorest population in Khyber Pakhtunkhwa province, Pakistan. International Journal for Equity in Health. 2024 Sep 23;23(1):190.

30.	Carlin CS, Christianson JB, Keenan P, Finch M. Chronic Illness and Patient Satisfaction. Health Services Research. 2012 Dec;47(6):2250–72.

31.	Omer S, Pan M, Ali S, Shukar S, Fang Y, Yang C. Perceptions of pharmacists towards drug shortages in the healthcare system of Pakistan and its impact on patient care: findings from a cross-sectional survey. BMJ Open. 2021 Dec 1;11(12):e050196.

32.	Le N, Groot W, Tomini S, Tomini F. Health insurance and patient satisfaction: Evidence from the poorest regions of Vietnam [Internet]. United Nations University - Maastricht Economic and Social Research Institute on Innovation and Technology (MERIT); 2018. Report No.: 2018–040. Available from: https://EconPapers.repec.org/RePEc:unm:unumer:2018040

Appendix

Appendix 1

Emojis associated to each modality of the satisfaction questions
