# Supplementary material for: Examining individual, health service, and experience of care determinants of patients’ satisfaction - A cross-sectional facility-based study in KP, Pakistan
Source: PLoS One. 2026 May 27;21(5):e0346788. doi: 10.1371/journal.pone.0346788 (PMC13215502; doi:10.1371/journal.pone.0346788)
Supplement: Attachment — Submitted filename: Rebuttal letter_mda clean.docx [file pone.0346788.s006.docx]

Dear Dr. Petitfour,

Thank you for submitting your manuscript to PLOS ONE.

After careful consideration, we feel that it has merit but does not fully meet PLOS ONE’s publication criteria as it currently stands and requires a "major revision" before the journal can further consider this manuscript. Therefore, we invite you to submit a revised version of the manuscript that addresses the points raised during the review process. In addition, kindly provide all survey/study tools if you agree to resubmit the manuscript.

Please submit your revised manuscript by Jul 19 2025 11:59PM. If you will need more time than this to complete your revisions, please reply to this message or contact the journal office at [plosone@plos.org](mailto:plosone@plos.org). When you're ready to submit your revision, log on to <https://www.editorialmanager.com/pone/> and select the 'Submissions Needing Revision' folder to locate your manuscript file.

Please include the following items when submitting your revised manuscript:

- A rebuttal letter that responds to each point raised by the academic editor and reviewer(s). You should upload this letter as a separate file labeled 'Response to Reviewers'.
- A marked-up copy of your manuscript that highlights changes made to the original version. You should upload this as a separate file labeled 'Revised Manuscript with Track Changes'.
- An unmarked version of your revised paper without tracked changes. You should upload this as a separate file labeled 'Manuscript'.

If you would like to make changes to your financial disclosure, please include your updated statement in your cover letter. Guidelines for resubmitting your figure files are available below the reviewer comments at the end of this letter.

If applicable, we recommend that you deposit your laboratory protocols in protocols.io to enhance the reproducibility of your results. Protocols.io assigns your protocol its own identifier (DOI) so that it can be cited independently in the future. For instructions see: [https://journals.plos.org/plosone/s/submission-guidelines#loc-laboratory-protocols](https://track.editorialmanager.com/CL0/https:%2F%2Fjournals.plos.org%2Fplosone%2Fs%2Fsubmission-guidelines%23loc-laboratory-protocols/1/010f01973a942a42-5ce9b14c-de27-40cc-9767-48b85797a8f3-000000/hxwKAEFfz9fyki9hvY7WG9l6eJvNNeTzzGIJgdLPARo=214). Additionally, PLOS ONE offers an option for publishing peer-reviewed Lab Protocol articles, which describe protocols hosted on protocols.io.

Read more information on sharing protocols at

<https://plos.org/protocols?utm_medium=editorial-email&utm_source=authorletters&utm_campaign=protocols>.

We look forward to receiving your revised manuscript.

Kind regards,

Dr. Syed Khurram Azmat, PhD, MPH, MD

Academic Editor

PLOS ONE

 Journal Requirements:

When submitting your revision, we need you to address these additional requirements.

1. Please ensure that your manuscript meets PLOS ONE's style requirements, including those for file naming. The PLOS ONE style templates can be found at

<https://journals.plos.org/plosone/s/file?id=wjVg/PLOSOne_formatting_sample_main_body.pdf> and
[https://journals.plos.org/plosone/s/file?id=ba62/PLOSOne_formatting_sample_title_authors_affiliations.pdf](https://track.editorialmanager.com/CL0/https:%2F%2Fjournals.plos.org%2Fplosone%2Fs%2Ffile%3Fid=ba62%2FPLOSOne_formatting_sample_title_authors_affiliations.pdf/1/010f01973a942a42-5ce9b14c-de27-40cc-9767-48b85797a8f3-000000/EW6Iys_9BKyEnw-JJVgTeO1po_AnziISTfkA34ddKN4=214)

2. Please include a complete copy of PLOS’ questionnaire on inclusivity in global research in your revised manuscript. Our policy for research in this area aims to improve transparency in the reporting of research performed outside of researchers’ own country or community. The policy applies to researchers who have travelled to a different country to conduct research, research with Indigenous populations or their lands, and research on cultural artefacts. The questionnaire can also be requested at the journal’s discretion for any other submissions, even if these conditions are not met. Please find more information on the policy and a link to download a blank copy of the questionnaire here: <https://journals.plos.org/plosone/s/best-practices-in-research-reporting>. Please upload a completed version of your questionnaire as Supporting Information when you resubmit your manuscript.

3. Thank you for stating in your Funding Statement:

[Open Access funding enabled and organized by Projekt DEAL. We gratefully acknowledge financial support as part of a scientific collaboration with the KfW Development Bank (“Scientific implementation research on outpatient-department (OPD) services in the social health protection initiative, Khyber Pakhtunkhwa (KP) province and the Gilgit Baltistan (GB) area”).].

Please provide an amended statement that declares *all* the funding or sources of support (whether external or internal to your organization) received during this study, as detailed online in our guide for authors at <http://journals.plos.org/plosone/s/submit-now>.  Please also include the statement “There was no additional external funding received for this study.” in your updated Funding Statement.

Please include your amended Funding Statement within your cover letter. We will change the online submission form on your behalf.

4. Please expand the acronym “KfW” (as indicated in your financial disclosure) so that it states the name of your funders in full.

This information should be included in your cover letter; we will change the online submission form on your behalf.

5. Thank you for stating the following in your manuscript:

[Open Access funding enabled and organized by Projekt DEAL. We gratefully acknowledge financial support as part of a scientific collaboration with the KfW Development Bank (“Scientific implementation research on outpatient-department (OPD) services in the social health protection initiative, Khyber Pakhtunkhwa (KP) province and the Gilgit Baltistan (GB) area”).]

We note that you have provided funding information that is currently declared in your Funding Statement. However, funding information should not appear in the Acknowledgments section or other areas of your manuscript. We will only publish funding information present in the Funding Statement section of the online submission form.

Please remove any funding-related text from the manuscript and let us know how you would like to update your Funding Statement. Currently, your Funding Statement reads as follows:

[Open Access funding enabled and organized by Projekt DEAL. We gratefully acknowledge financial support as part of a scientific collaboration with the KfW Development Bank (“Scientific implementation research on outpatient-department (OPD) services in the social health protection initiative, Khyber Pakhtunkhwa (KP) province and the Gilgit Baltistan (GB) area”).]

Please include your amended statements within your cover letter; we will change the online submission form on your behalf.

6. When completing the data availability statement of the submission form, you indicated that you will make your data available on acceptance. We strongly recommend all authors decide on a data sharing plan before acceptance, as the process can be lengthy and hold up publication timelines. Please note that, though access restrictions are acceptable now, your entire data will need to be made freely accessible if your manuscript is accepted for publication. This policy applies to all data except where public deposition would breach compliance with the protocol approved by your research ethics board. If you are unable to adhere to our open data policy, please kindly revise your statement to explain your reasoning and we will seek the editor's input on an exemption. Please be assured that, once you have provided your new statement, the assessment of your exemption will not hold up the peer review process.

7. Please amend the manuscript submission data (via Edit Submission) to include author Zohaid KHAN.

There is no Zohaid Khan as an author, only a Zohaib Khan. The list on the submission data is correct, the type was in the initial manuscript, that has been corrected since then.

8. Please amend your authorship list in your manuscript file to include author Zohaib KHAN.

Zohaib Khan is in the author list in our manuscript. There might have been a confusion because there was initially a typo in his first name, that we amended (it was Zohaid, while his first name in Zohaib).

9. We note that Figure 1 in your submission contain map images which may be copyrighted. All PLOS content is published under the Creative Commons Attribution License (CC BY 4.0), which means that the manuscript, images, and Supporting Information files will be freely available online, and any third party is permitted to access, download, copy, distribute, and use these materials in any way, even commercially, with proper attribution. For these reasons, we cannot publish previously copyrighted maps or satellite images created using proprietary data, such as Google software (Google Maps, Street View, and Earth). For more information, see our copyright guidelines: [http://journals.plos.org/plosone/s/licenses-and-copyright](http://track.editorialmanager.com/CL0/http:%2F%2Fjournals.plos.org%2Fplosone%2Fs%2Flicenses-and-copyright/1/010f01973a942a42-5ce9b14c-de27-40cc-9767-48b85797a8f3-000000/5iBQ9giMVBu6B56fE3t20UTEZxyzo7mg0haa1maZ9Yc=214).

We require you to either (1) present written permission from the copyright holder to publish these figures specifically under the CC BY 4.0 license, or (2) remove the figures from your submission:

1. You may seek permission from the original copyright holder of Figure 1 to publish the content specifically under the CC BY 4.0 license.

We recommend that you contact the original copyright holder with the Content Permission Form (<http://journals.plos.org/plosone/s/file?id=7c09/content-permission-form.pdf>) and the following text:

“I request permission for the open-access journal PLOS ONE to publish XXX under the Creative Commons Attribution License (CCAL) CC BY 4.0 (<http://creativecommons.org/licenses/by/4.0/>). Please be aware that this license allows unrestricted use and distribution, even commercially, by third parties. Please reply and provide explicit written permission to publish XXX under a CC BY license and complete the attached form.”

Please upload the completed Content Permission Form or other proof of granted permissions as an "Other" file with your submission.

In the figure caption of the copyrighted figure, please include the following text: “Reprinted from [ref] under a CC BY license, with permission from [name of publisher], original copyright [original copyright year].”

2. If you are unable to obtain permission from the original copyright holder to publish these figures under the CC BY 4.0 license or if the copyright holder’s requirements are incompatible with the CC BY 4.0 license, please either i) remove the figure or ii) supply a replacement figure that complies with the CC BY 4.0 license. Please check copyright information on all replacement figures and update the figure caption with source information. If applicable, please specify in the figure caption text when a figure is similar but not identical to the original image and is therefore for illustrative purposes only.

The following resources for replacing copyrighted map figures may be helpful:

USGS National Map Viewer (public domain): <http://viewer.nationalmap.gov/viewer/>
The Gateway to Astronaut Photography of Earth (public domain):

<http://eol.jsc.nasa.gov/sseop/clickmap/>
Maps at the CIA (public domain):

<https://www.cia.gov/library/publications/the-world-factbook/index.html> and

<https://www.cia.gov/library/publications/cia-maps-publications/index.html>
NASA Earth Observatory (public domain): <http://earthobservatory.nasa.gov/>

Landsat: <http://landsat.visibleearth.nasa.gov/>

USGS EROS (Earth Resources Observatory and Science (EROS) Center) (public domain): [http://eros.usgs.gov/#](http://eros.usgs.gov/)
Natural Earth (public domain): [http://www.naturalearthdata.com/](http://track.editorialmanager.com/CL0/http:%2F%2Fwww.naturalearthdata.com%2F/1/010f01973a942a42-5ce9b14c-de27-40cc-9767-48b85797a8f3-000000/64i0EQS1e75RTvyGTEUkbMiMBBdTf-4Gc3uLlQ8RCJU=214)

We deleted Figure 1 of the manuscript

10. Please include captions for your Supporting Information files at the end of your manuscript, and update any in-text citations to match accordingly. Please see our Supporting Information guidelines for more information: <http://journals.plos.org/plosone/s/supporting-information>.

Reviewers' comments:

Reviewer's Responses to Questions

**Comments to the Author**

1. Is the manuscript technically sound, and do the data support the conclusions?

The manuscript must describe a technically sound piece of scientific research with data that supports the conclusions. Experiments must have been conducted rigorously, with appropriate controls, replication, and sample sizes. The conclusions must be drawn appropriately based on the data presented.

Reviewer #1: Yes

Reviewer #2: No

2. Has the statistical analysis been performed appropriately and rigorously?

Reviewer #1: Yes

Reviewer #2: No

3. Have the authors made all data underlying the findings in their manuscript fully available?

The [PLOS Data policy](http://track.editorialmanager.com/CL0/http:%2F%2Fwww.plosone.org%2Fstatic%2Fpolicies.action%23sharing/1/010f01973a942a42-5ce9b14c-de27-40cc-9767-48b85797a8f3-000000/AcS7Yi5g8TRn1nPE9XvhaGRXbuJGW7t0h4xqG286Tx4=214) requires authors to make all data underlying the findings described in their manuscript fully available without restriction, with rare exception (please refer to the Data Availability Statement in the manuscript PDF file). The data should be provided as part of the manuscript or its supporting information, or deposited to a public repository. For example, in addition to summary statistics, the data points behind means, medians and variance measures should be available. If there are restrictions on publicly sharing data—e.g. participant privacy or use of data from a third party—those must be specified.

Reviewer #1: Yes

Reviewer #2: No

4. Is the manuscript presented in an intelligible fashion and written in standard English?

PLOS ONE does not copyedit accepted manuscripts, so the language in submitted articles must be clear, correct, and unambiguous. Any typographical or grammatical errors should be corrected at revision, so please note any specific errors here.

Reviewer #1: Yes

Reviewer #2: Yes

5. Review Comments to the Author

Please use the space provided to explain your answers to the questions above. You may also include additional comments for the author, including concerns about dual publication, research ethics, or publication ethics. (Please upload your review as an attachment if it exceeds 20,000 characters)

Reviewer #1: Dear Authors,

Congratulations on the successful submission of your manuscript titled. I commend you for your hard work and dedication in bringing this research to fruition.

With minor edits and clarifications, this paper is ready for publication. The research is original, well-designed, and has strong potential policy relevance for healthcare delivery in LMIC contexts.

A general comment is to use smaller sentences; some sentences, especially in introduction and discussion sections are very long, these can be split into smaller sentences for better readability. Please see additional comments/suggestions in the attached document and address them for resubmission.

Best of luck and warm regards,

Reviewer #2: It is a good study that attempted to study determinants of patient satisfaction in the Khyber Pakhtunkhwa province of Pakistan. However, it suffers some major limitations and ambiguities. The detailed feedback is presented below

Title: "What affects a patient’s satisfaction with healthcare?"

• The title is currently too broad. Consider specifying the determinants being assessed — for example, whether they are related to health system factors, patient characteristics, or both. The current phrasing is vague and lacks focus.

We thank the reviewer for this feedback. Indeed, we initially chose a generic title, but rephrased it in the amended manuscript.

Previous title What affects a patient’s satisfaction with healthcare?

New title Examining individual, health service, and experience of care determinants of patients’ satisfaction - a cross-sectional facility-based study in KP, Pakistan

Abstract:
• Clearly state the objective of the study at the outset.

• Specify the tool or instrument used to assess satisfaction (e.g., “A 13-item scale was used to measure patient satisfaction…”).

• To improve clarity, organize the results by factor type — for instance, patient characteristics versus systemic or facility-level factors. Currently, these are mixed together.

• The sentence “being insured by an inpatient-focused insurance was significantly associated with satisfaction” requires clarification. Since the survey targeted patients exiting outpatient services, it's unclear how inpatient insurance coverage applies here.

We reviewed the abstract according to most of the feedback provided by the reviewer, and think it does include its clarity on the study rationale and method. Due to a strong word constraint, we could explain here why being insured for inpatient services can be associated to a higher satisfaction, but we provide these reasons only in the presentation of the exposure variables in the manuscript.

Abstract (previous version)

Health systems aim to meet populations’ needs and to spur their satisfaction, requiring to first understand what drives this satisfaction, whether these drivers come from the health system or not. In this empirical study, we use survey data from 666 patients exiting outpatient services of 60 public and 62 private health facilities in four districts of the province of Khyber Pakhtunkhwa, Pakistan.

We asked patients questions about their socio-economic situation and their satisfaction regarding several aspects of the healthcare they just received. We then investigated elements of this experience (respect of privacy, out-of-pocket expenditures, availability of drugs). First, we implemented a Principal Component Analysis to compute a composite Patient Satisfaction score. Second, we used this score as the outcome in a linear model to identify its determinants.

We found a generally high level of satisfaction regarding outpatient services within the sample, with an average satisfaction score of 76.37 out of 100.). We did not find any association between satisfaction and the type of facility. Being a female patient and being insured by an inpatient-focused insurance were significantly associated with satisfaction (respectively negatively and positively). The variables that affected satisfaction the most were the characteristics of the healthcare experience, namely: privacy (positively) availability of drugs (positively) and cost of healthcare (negatively).

Our results highlight the importance of considering the specific needs of a subpopulation (e.g. female patients) to provide them with appropriate care and to ensure the respect of core aspects of healthcare experiences, in all types of facility.

Abstract (amended version)

Health systems aim to meet populations’ needs and to spur their satisfaction, requiring to first understand what drives this satisfaction, whether these drivers come from the health system or not. In this empirical study, we use survey data from 666 patients exiting outpatient services of 60 public and 62 private health facilities in four districts of the province of Khyber Pakhtunkhwa, Pakistan.

The survey tool assessed patients’ socio-economic status, satisfaction with health service provision, and experiences of care (respect of privacy, out-of-pocket expenditures, availability of drugs). First, we synthesized the 17 items relating to satisfaction into a 11-item scale Patient Satisfaction score, relying on a Principal Component Analysis. Second, we used this score as the outcome in a linear model to identify its determinants.

We found a generally high level of satisfaction regarding outpatient services within the sample, with an average satisfaction score of 76.37 out of 100. We did not find any association between satisfaction characteristics of the environment (type of facility, district). Women displayed significantly lower (-16%) and insured patients significantly higher (+6%) satisfaction scores. Indicators of healthcare experiences were found to be the strongest determinants of satisfaction, namely: satisfaction scores were 12% higher when privacy was respected, 5% higher when prescribed drugs were available and they also varied with the amount of healthcare expenses (positively and then negatively).

Our results highlight the importance of designing policies that address the needs of specific population sub-groups and of ensuring abidance to core aspects of healthcare delivery, as reflected in patients’ experiences, across all facility types.

Data Availability:

• The statements are contradictory: first, it says “No – some restrictions will apply”, then it claims data will be publicly available. Please clarify the actual data availability status and correct inconsistencies.

Data will be made publicly available.

Introduction:
• Include studies conducted in Khyber Pakhtunkhwa (KP) province to enhance contextual relevance. Consider citing other papers:

o <https://journalofkcd.com/kcd/article/view/25>

o <https://jpma.org.pk/index.php/public_html/article/view/9990>

We thank the reviewer for this recommendation, which we followed by adding several references, all pertaining to empirical studies conducted in KP. We have enriched the literature review by including studies on patient satisfaction in Pakistan and more specifically in KP, such as …

Idrees M, Ali Shah SN, Iqbal W, Bazaz SA, Bukhari F. Analysis Of Consultation Length In Khyber Pakhtunkhwa, Pakistan. J Ayub Med Coll Abbottabad. 2021;33(3):424–30.

Wasim M, Rehman K, Paracha M, Sarfaraz J, Noor SM. Patient Satisfaction Level and its Various Determinants in a Tertiary Care Hospital of Peshawar, Pakistan: A Cross-Sectional Study: Patient Satisfaction Level in a Tertiary Care Hospital. Pakistan Journal of Health Sciences. 2025 Feb 28;247–52.

Mushtaq M, Shah K, Khattak YR, Khattak N, Farheen H, Akhtar S. Satisfaction of patients in government medical teaching institutes of Peshawar Khyber Pakhtunkhwa, Pakistan: a cross-sectional analytical study. Journal of the Pakistan Medical Association. 2024 Mar 20;74(4):701–5.

Hassan MZU, Hameed Z, Hussan J, Ahmad O, Haris M, Shah HU, et al. AN ASSESSMENT OF PATIENT SATISFACTION IN OPD SETUPS OF KHYBER TEACHING HOSPITAL MTI PESHAWAR. JOURNAL OF KHYBER COLLEGE OF DENTISTRY. 2023 Sep 30;13(3):23–8.

• The rationale for the study is currently weak. Clearly highlight specific gaps in the existing literature and explain how this study addresses them. The current explanation is generic and does not clearly demonstrate the study’s unique contribution.

In the same paragraph as the literature review, we made clearer what is already known from the existing literature, the remaining gaps and how our study brings additional evidence to the field

Former version of paragraph 4 of the introduction

In Pakistan empirical evidence on patient satisfaction in the context of outpatient care is so far limited. Surveys of outpatients receiving care at a tertiary hospital of Karachi identified patient dissatisfaction with aspects of the patient-provider interaction, such as insufficient time to explain and answer questions and lack of privacy and respectfulness (15,16). Interviews with outpatients at a public diabetes clinic in Punjab province found positive patient satisfaction to be associated with competent provider communication, and was generally higher among patients who were female, uneducated, or employed (17). Studies conducted at hospitals and tertiary care centres found that both outpatients and inpatients were generally more satisfied with the care received from the private sector compared with public sector providers (18,19). While positive patient satisfaction with private providers was associated with prompt service delivery, satisfaction with private providers was associated with empathy for patients’ problems and needs (20).

Amended version of paragraph 4 of the introduction (page 3/4)

In Pakistan, and specifically in the Khyber Pakhtunkhwa (KP) province, our study setting, the recent empirical evidence on patient satisfaction has provided some answers on the level of satisfaction in health facilities. Yet, several gaps remain. First, most studies have focused on tertiary facilities, reflecting satisfaction with inpatient care (16,17) rather than outpatient care (18). This is in spite of the fact that from the health system perspective, strengthening the primary care level of care is crucial to ensure UHC. Hence, it is crucial to understand patients’ perspectives about their experience with primary level facilities and their satisfaction with care received at this level. Second, some studies have linked higher patient satisfaction to characteristics of patients or their experience with receiving care, or to facility characteristics, but to our knowledge, none has considered both. In other Pakistani settings than KP, patient satisfaction in outpatient services was found to be associated with aspects of the patient-provider interaction, such as time to explain and answering questions, ensuring privacy and respectfulness (19–21). Individual characteristics like being female, uneducated, or employed were also found to be associated with higher satisfaction (21). Other studies, conducted among diverse hospitals and tertiary care centres, considered facility characteristics and found that patients were generally more satisfied with the care received in the private sector compared to the public sector (22,23). In synthesis, our review of existing literature identifies the need to conduct analyses of patients’ satisfaction that account for multiple determinants simultaneously, generating more comprehensive evidence for policy.

• The phrase “…identifying determinants of patients’ satisfaction…” should be more specific. Explicitly mention whether these determinants include demographic, financial, structural, or service-related factors.

We worked on the rationale of the study and on the study objective to make it more explicit, precise and concrete.

Former version of the paragraph 4 of the introduction

Our study aims at identifying determinants of patients’ satisfaction with outpatient care services in the province of Khyber Pakhtunkhwa, Pakistan. Our study adds to the existing literature by examining patients’ satisfaction in a remote region of the country, relying on a large sample of patients, drawn from a large sample of primary level facilities. It also uses a more comprehensive measure than what has been used in the country in the past, considering several aspects of the experience of care in the outpatient setting.

Amended version of the paragraph 4 of the introduction (page 4)

There is a need for taking into consideration several types of determinants simultaneously to provide more comprehensive evidence.

Our study aims at identifying determinants of patients’ satisfaction with outpatient care services at the primary care level in KP accounting for three sets of exposure variables: individual patient characteristics; health facility infrastructure; and patients’ experience with health service delivery. Our study adds to the existing literature by examining patients’ satisfaction in a remote region of the country, relying on a large sample of patients, drawn from a diverse sample of primary level facilities of various types, enabling us to include a more diverse set of exposure variables than prior studies.

Methods:
• Clearly state the study design and the rationale for the selection of districts. Was the sampling random, purposive, or part of a larger study?

The selection of the districts was determined by the overall implementation research project within which our study is embedded. Our team is engaged in the prospective evaluation of an upcoming health insurance project targeting primary care services, which is to be launched in the four districts. As such the four districts were selected in agreement with the local government for data collection. More information can be found [here](https://blogs.fau.de/inspirepakistan/). We added a sentence to add this point in the manuscript (Methods, Study setting and sampling).

Former version of the paragraph 4 of the Methods, Study setting and sampling

Our study area constituted four districts of Khyber Pakhtunkhwa province in Pakistan: Chitral, Kohat, Malakand and Mardan (see Figure 1).

Amended version of the paragraph 4 of the Methods, Study setting and sampling (page 4/5)

We collected data in four districts in the Khyber Pakhtunkhwa province of Pakistan: Chitral, Kohat, Malakand and Mardan. These districts were selected in agreement with the local health authorities as the target of an upcoming health insurance program targeting primary healthcare services. No health insurance targeting primary care was yet active at the time of data collection. (see Figure 1).

Define the eligibility criteria for participants — such as age, type of medical condition, or type of healthcare service utilized.

We included all adult patients exiting the general outpatient services who were consenting to take part to the study, irrespective of their condition. Children or disabled patients that could not respond to the questionnaire on their own were excluded.

The inclusion and exclusion criteria are mentioned in the Methods section, in the Sampling subsection.

Former version of the paragraph 4 of the Methods section, Sampling subsection

Visiting each sampled provider, we conducted exit interviews with patients exiting the premises after having attended outpatient care services. Our teams were stationed with each provider for one day with the aim to reach eight patients per provider. We included only adult patients. Children or disabled patients that could not respond to the questionnaire on their own were excluded.

Amended version of the paragraph 3 of the Methods section, Sampling and interview setting subsection (page 6/7)

At each sampled primary healthcare facility, we conducted exit interviews with patients exiting the facility premises after having attended outpatient care services. Our teams were stationed at each facility for one day. The surveyors introduced themselves, and the study to the patients, and asked them for their consent to participate. They also asked a few questions to check their eligibility. At each facility, on a given day, we included any adult patient exiting general outpatient services who consented to take part to the study, irrespective of their condition. Children or disabled patients that could not respond to the questionnaire on their own were excluded. After consenting to the interview, enumerators sat in a quiet and isolated location in or around the facility with consenting patients and administered the interview, lasting about 20 minutes. Enumerators had a target of interviewing eight patients per provider. We sat a target of reaching a minimum of eight interviews per facility, but enumerators could interview more in larger facilities with greater patient attendance.

• Explain how the sample size was determined. If this is a secondary analysis, indicate whether the existing sample was sufficient for your study objectives.

We have included the following paragraph in the methods section (page 6) followed by the paragraph indicated already above illustrating respondent selection:

The sample size was constrained by the overall number of facilities providing services in the four districts and by the objective of the overall study hosting the analysis presented in this paper. The overall objective is to monitor the implementation of a health insurance scheme in the study area. Hence, our sample included a representative sample of all healthcare providers, public and private, providing primary care services across the four districts. We included all DHQs, THQs, and RHQs (as people also seek primary outpatient services at this level) - identified as hospitals - and a random sample stratified by district of all BHUs to ensure district representation. Similarly, we included a random sample of all private providers stratified by district, not by facility type due to absence of information. Health facility lists, setting the basic sampling frame, were handed to the research team by the local health authorities.

Considering a total of 122 facilities and a minimum of 8 exit interviews per facilities, we would have expected an effective sample of 976 observations, enough to detect a proportion of 80% of satisfied patients according to the WHO sample-size-estimator with the following parameters:

| **Level of Confidence Measure** | 1,96 |
| --- | --- |
| **Margin of Error (MOE)** | 0,05 |
| **Baseline levels of the indicators** | 0,8 |
| **Design effect (Deff)** | 1,5 |
| **Expected Response Rate** | 0,8 |
| **Number of age/sex Estimates** | 2 |

(final sample size required according to WHO, 922)

• Describe the interview setting, duration, and who conducted the interviews.

We added a sentence in the Sampling and interview setting subsection in Methods section:

Previous version of the paragraph

Visiting each sampled provider, we conducted exit interviews with patients exiting the premises after having attended outpatient care services. Our teams were stationed with each provider for one day with the aim to reach eight patients per provider. We included only adult patients. Children or disabled patients that could not respond to the questionnaire on their own were excluded.

Amended version of the paragraph (page 6/7)

At each sampled primary healthcare facility, we conducted exit interviews with patients exiting the facility premises after having attended outpatient care services. Our teams were stationed at each facility for one day. The surveyors introduced themselves, and the study to the patients, and asked them for their consent to participate. They also asked a few questions to check their eligibility. At each facility, on a given day, we included any adult patient exiting general outpatient services who consented to take part to the study, irrespective of their condition. Children or disabled patients that could not respond to the questionnaire on their own were excluded. After consenting to the interview, enumerators sat in a quiet and isolated location in or around the facility with consenting patients and administered the interview, lasting about 20 minutes. Enumerators had a target of interviewing eight patients per provider. We sat a target of reaching a minimum of eight interviews per facility, but enumerators could interview more in larger facilities with greater patient attendance.

• Line 119–120: Clarify whether these variables were additional to the 21 satisfaction items or overlapping.

We thank the reviewer for their vigilance and clarified this sentence. Please see below the former and new version of this paragraph.

• Line 124–125: The phrase “capacity to explain…” is unclear and should be reworded for precision. Similarly, ‘rate of the quality of care’?

We thank the reviewer for their vigilance and clarified this sentence. Please see below the former and new version of this paragraph.

• Line 126–127: “It further included eight binary satisfaction variables…” needs clarification. If these overlap with the previously mentioned variables, reorganize and integrate this information earlier for coherence.

Indeed, there was repetition in the presentation of the variables, and we are sorry for this. We reworked the paragraph and present an amended and clearer version of it.

Former version of the Questionnaire subsection in Methods section

The second section consisted of 21 survey questions collecting information on each patient’s satisfaction with the healthcare they had just received. The first four questions captured aspects of patients’ general satisfaction with the healthcare visit (e.g. whole care-seeking experience, likelihood to recommend the provider). The following 17 questions were related to specific aspects of the healthcare encounter (e.g., respect shown by provider, respect of confidentiality). A total of nine questions used a 5-item Likert scale to capture responses to questions phrased as: “How satisfied were you with <SPECIFIC HEALTHCARE ASPECT>?”. Scaled responses in the questionnaire were represented by five emoticons representing level of satisfaction ranging from “not smiling at all” to “much smiling” as depicted in Appendix 1, which was shown to the respondents to facilitate their understanding of the scale. The remaining eight questions were asked in a binary way (“Were you satisfied with <SPECIFIC HEALTHCARE ASPECT>?”), with responses recorded as “yes” or “no”. The set of four general satisfaction variables included 5-item Likert scale variables asking about the satisfaction regarding: care-seeking experience, rate of the quality of care, rate of the quality of care in relation to the price and likelihood to recommend this facility to others. The set of 17 variables for satisfaction regarding specific dimensions of the healthcare experience included nine 5-item Likert scale variables asking about the satisfaction regarding: respect shown by provider, confidentiality level, capacity to explain shown by provider, capacity to take time for questions shown by provider, capacity to take time shown by provider, waiting time, distance, adequacy of building, adequacy of staff. It included further included eight binary satisfaction variables regarding: cleanliness, waiting room, smell, infrastructure cleanliness, access to clean toilet, access to clean water, access to soap, disability access.

Amended version of the Questionnaire subsection in Methods section (page 7)

The second section included 20 questions collecting information on each patient’s satisfaction with the healthcare they had just received. Within these 20 questions, the first four ones referred to patients’ general satisfaction with the healthcare visit. Responses were captured on a 5-item Likert scale on: 1) the care-seeking experience; 2) the quality of care during this experience; 3) the quality of care in relation to the price; and 4) the likelihood to recommend this facility to others. The remaining 16 questions related to specific aspects of the healthcare encounter (e.g., respect shown by provider, respect of confidentiality). Nine out of these 16 questions also relied on a 5-item Likert scale to capture responses to questions phrased as: “How satisfied were you with <SPECIFIC HEALTHCARE ASPECT>?”. Healthcare aspects included respect shown by provider, confidentiality level, capacity to explain diagnosis and potential treatments shown by provider, capacity to take time for questions shown by provider, capacity to take time shown by provider, waiting time, distance, adequacy of building, adequacy of staff. All scaled responses were represented by five emoticons representing level of satisfaction ranging from “not smiling at all” to “much smiling” as depicted in Appendix 1, shown to the respondents to facilitate their understanding of the scale. The remaining seven questions elicited a simple binary response, YES vs NO, to the question “Were you satisfied with <SPECIFIC HEALTHCARE ASPECT>?”. The concerned health aspects were cleanliness, waiting room, smell, access to clean toilet, access to clean water, access to soap, disability access.

Construction of composite index

• Line 144: Explain why four general satisfaction variables were excluded.

We excluded the four general satisfaction questions from the construction of the composite score because we considered they had a different status than the questions focusing on satisfaction regarding specific aspects of the healthcare experience. We now explain this choice in the manuscript (see below for the former and new version of this paragraph).

Additionally, we implemented the first stage including these four variables in the first stage, and then implemented the second stage with the alternative outcome. We obtained the same results in terms of significance and signs of our coefficients, confirming our main results and conclusions.

• Line 145: Explain the reason for removal of variables with clear statistical criteria (e.g., low factor loadings or communalities). Mention the cut-off values used.

We thank the reviewer for noting the unclear explanation on this. We explained the reason for excluding 4 variables, linked to the too high proportion of missing values, which would have restricted the sample for the whole analysis.

In addition, after discussion with the field teams, these missing values were due to “non applicable” cases: many respondents mentioned not needing toilets as it is only an outpatient clinic, and so did not answer the corresponding questions. Many also mentioned not needing a specific access for disable people, hence they simply did not pay attention to its availability. This reinforced our decision in excluding the variables because it indicated that the high proportion of missing value was due to the respondents not seeing high applicability of the questions.

Please see below for the former and amended version of the corresponding subsection

• Line 148: Clarify the rationale for using normalized eigenvalues as satisfaction scores instead of first principal component. The eigenvalues tell us the variance component of PCA.

We thank reviewers for their careful reading of the manuscript, the eigenvalue is indicated as such in Table 1 (4.99), with the corresponding proportion of variance explained (41%).

We provide the coefficient associated to each variable in the first component in Table 1 also. We normalized only the scores obtained with this first component. This allowed us to have values ranging between 0 and 100, which eases the interpretation.

• Overall, the section on satisfaction item lack conceptual clarity. Whether the tool was validated or whether the 17 satisfaction items comprehensively captures all the dimensions of the satisfaction construct. A conceptual framework or mapping of domains would enhance clarity.

We reworked carefully this paragraph, and hope it relates better the way we proceeded to go from a raw set of 20 questions to a synthetic composite Satisfaction Score.

In addition, we also added a sentence mentioning our checks regarding the reliability of our Satisfaction construct. Its Alpha score is 0.8525, which is highly satisfying, and there is no removal of any item that increases it. We then kept all items for our Satisfaction score.

Previous version of the “Construction of the composite satisfaction Index” subsection

During the first stage, we synthesized the information captured by all 21 questions related to satisfaction into one measure. Following the literature, we considered only aspects of satisfaction at this stage and did not yet consider actual elements of the healthcare experience (21). We then computed the correlation matrix for these 21 satisfaction variables to check that they were all significantly and positively correlated. Next, we conducted a Principal Component Analysis (PCA) using only the 17 variables measuring satisfaction with specific dimensions of the healthcare experience (i.e., we excluded the four variables on general satisfaction). Six variables were then removed because of missing values or limited explanatory power for the first principal component. Ultimately, the final PCA model included 11 of the initial 21 variables (see Table 1). In this model, the first component explained 41% of the total variability. We normalized eigenvalues to range from 0 to 100 to ease interpretation. Hereafter, we refer to the derived composite variable as the Satisfaction Score.

Amended version (page 8)

During the first stage, we synthesized the information captured by all 20 questions related to satisfaction into one measure. Following the literature, we considered only aspects of satisfaction at this stage and did not yet consider actual elements of the healthcare experience (25). We then computed the correlation matrix for the 20 satisfaction variables to check that they were all significantly and positively correlated. Next, we conducted a Principal Component Analysis (PCA) using only the 16 variables measuring satisfaction with specific dimensions of the healthcare experience (i.e., we excluded the four variables on general satisfaction). Indeed, we considered that introducing them in the composite index would blur the interpretation of the components, as the ultimate would include both general and specific variables. In addition, global satisfaction is likely to be mechanically correlated to the specific correlation items, as it is driven by them. Four variables were further removed because they exhibited more than 20% of missing values. Ultimately, the final PCA model included 12 of the initial 20 variables (see Table 1). The first component explained 41% of the total variability. We further tested the reliability of our measure and the correlation of each item with it by measuring the Alpha Cronbach score (=0.8525) and the Alpha score when removing each item one by one. We kept all items. We normalized the scores from this first component to range from 0 to 100 to ease interpretation. Hereafter, we refer to the derived composite variable as the Satisfaction Score.

Statistical Analysis:

• Patients were sampled equally across facilities, despite variations in patient volume. Explain why weighting was not used to adjust for this imbalance.

We are grateful for this comment.

We recognize that sampling according to patient volume might have been preferable, however in the absence of a reliable health management information system, this was not feasible. It would have required an excessive effort, first counting all patient encounters recorded manually in the facility records. Moreover, most studies exploring patients’ satisfaction via means of exit interview apply similar methodologies, with a fixed number of interviews being implemented at every facility (for instance, Idrees et al, 2021; Mushtaq et al. 2024 in Pakistanese context).

We do acknowledge, however, the fact that we set only a minimum sample size per facilities and actively encouraged enumerators to interview more in larger facilities. Accordingly, to address the reviewer’s concern we have added a sentence in the methodological considerations to address this weakness (page 21).

We recognise the weakness arising from the fact that in the absence of reliable health management information systems, we could not sample respondents to account for facility size. Hence, our analysis is not weighted for facility size, resulting in an overrepresentation of views from patients from smaller healthcare facilities. Given the emphasis on primary healthcare, both of our analysis and of the upcoming schemes, this is not per se problematic.

Table 2:

• The stated rationale “Higher satisfaction in private facilities” needs clarification. It does not align clearly with the indicator ‘asset quintile’.

There was a typo, which is now corrected, it now mentions “higher satisfaction among people in the richest quintiles”.

• For the Sehat Plus Card variable:

o a) Re-confirm whether all residents of KP were eligible at the time of data collection or if it was limited to low-income groups. Per my understanding, SCP services for everyone in the KP who has a KP national identity card.

We confirm that both BISP (a national program) and the Sehat Plus Card (a program implemented in several districts in KP, including our four study districts) target the poorest in the population, and it was already the case at the time of data collection.

The BISP program initially targeted only the ultra-poor, then progressively its eligibility criteria were expanded; yet the program still only covers a share of the population, defined as the most vulnerable.

o b) Since the Sehat Card covers only inpatient services, please explain the relevance of this indicator when the focus is on outpatient care.

By introducing this variable, we wanted to test whether having the experience of health insurance, even with inpatient services only, could affect patients’ satisfaction with health services, possibly by shaping their overall experience of care.

Our theoretical postulation is that by being insured, they may gain familiarity with the healthcare system, and if they are reimbursed for some healthcare expenses related to inpatient care, this can make them globally more satisfied with the healthcare system in general, including outpatient cases.

Corresponding paragraph (page 12)

We included a binary variable indicating whether a patient was covered by the Sehat Plus Card, an inpatient health insurance program, and a binary variable indicating whether a patient was a beneficiary of the Benazir Income Support Programme (BISP), a wide-scale cash transfer social protection program. Both programs target the poorest households. The rationale was to test the hypothesis that being the beneficiary from any social protection program might increase familiarity with the local health system and by default also yield repercussions on one’s satisfaction with outpatient services.

Line 184:

• The dimension of ‘experience with healthcare seeking’ doesn’t seem to be studied comprehensively. It merely has only 3 indicators.

We understand the concern of the reviewer. However, we would like to point at the fact that we had to balance comprehensiveness against the feasibility of implementing the survey as an exit interview. We did not want to overload respondents and chose the key dimensions, in line with prior literature (Saleem et al., 2022; Akhta et al., 2023; Pratiwi et al., 2022, Khamis and Njau, 2014) and findings from our own qualitative work.

We have added a sentence to the methods section:

We include three items capturing patients’ experience of care, to remain focus on the key dimensions of service delivery, in line with prior literature and with findings from our own qualitative work.

Line 192–194:

• Strengthen the justification for cut-off used for out-of-pocket expenditure (OOPE).

Because several types of facilities do not charge fees when patients are in need or for specific reasons, we wanted to isolate this modality from the others.

In BHUs, patients are supposed to pay a fee of 10 PKR than includes the consultation and the drugs, if they are available. Therefore, we chose this cut-off to isolate this amount from higher ones.

The 800 PKR cut-off was chosen because it represents about more than one day of GDP per capita at the time of the survey (annual GDP per capita in PKR/365.25). Lastly, it represented the 20% in terms of OOPE, so it was a prevalent enough category to be introduced.

Please see below for the former and amended version of the paragraph.

• Provide more detail on the modeling approach: “We tested our model for both the logarithmic of OOPE and its square”.

It is likely that the effect of OOPE on satisfaction is not linear: for instance, that the payment of OOPE leads to higher satisfaction until a certain threshold, after which the association between OOPE and satisfaction becomes negative. We introduced both OOPE and their square to test this hypothesis.

Former version of the paragraph 4 of the Multivariate analysis of satisfaction score subsection

We also included the amount of medical out-of-pocket expenditures (OOPE) in logarithmic form to control for extreme values. We further tested potential non-linearity in the effect of OOPE using several categorical dummy variables: no OOPE, between 1 and 10 PKR, between 11 and 800 PKR and more than 800 PKR (all compared to the reference category of 10 PKR). Thresholds were chosen purposively to isolate patients who did not incur any OOPE, to distinguish between patients who paid the 10 PKR forfeit from patients paying higher OOPE, and 800 PKR to isolate the highest quintile of OOPE. We tested our model for both the logarithmic of OOPE and its square.

Amended version of the paragraph 4 of the Multivariate analysis of satisfaction score subsection (page 12/13)

Lastly, we included the amount of medical out-of-pocket expenditures (OOPE) in logarithmic form to control for extreme values. We further tested potential non-linearity in the effect of OOPE introducing the square of OOPE in our model. We also implemented an alternative way to capture the potential non-linear effects of OOPE, using several categorical dummy variables: no OOPE, between 1 and 10 PKR, between 11 and 800 PKR and more than 800 PKR (all compared to the reference category of 1 to10 PKR). Thresholds were chosen purposively to (i) isolate patients who did not incur any OOPE from all other patients; to (ii) distinguish between patients who paid the 10 PKR forfeit from patients paying higher OOPE; and to (i) isolate expenses which represent about more than one day of GDP per capita at the time of the survey (annual GDP per capita in PKR/365.25). This threshold of 800 PKR allows to isolate the highest quintile of OOPE.

Results:
• In Table 3, clarify what the values in parentheses represent — are these standard errors, or something else?

In Table 3, the values in parentheses are p-values, in Table 4 they are standard errors

Discussion:
• Not accounting for the type of service received by patient is also a limitation.

We agree with this comment and have added a sentence to this regard in the methodological consideration section.

Moreover, we recognise that not being able to control for diagnosis and/or type of service received might also be a major study limitation. However, our purpose was to provide an overall picture of patients’ satisfaction in the region and not to outline it by service/diagnosis type.

Overall feedback:

While some of the findings are interesting, the study suffer major limitations:

a) the rationale is weak as it does not specifically highlight the gaps in existing literature on the topic and how this particularly study addresses these gaps and add unique value to the existing body of knowledge;

We hope that the deepening of the literature, including what it already contains and the knowledge gaps now allows to better understand our novel contribution to the literature. To our knowledge, empirical studies in Pakistan have previously focused on one type of facility, and on one category of associated factors, while we attend to provide a more comprehensive perspective.

We have reworked the introduction to the pursue of this objective, better outlined the gap and the contribution.

b) the study revolves around patient satisfaction; however, the assessment of satisfaction lack conceptual underpinnings. Furthermore, the validity and reliability of the tool and the way construct is developed is unclear. Factor analysis would have been a better technique to develop the overall satisfaction construct;

We also reworked the parts of the Method section pertaining the construction of our Satisfaction measure, bringing in more clarity, and also new elements and checks that we had previously not put in the manuscript due to word limit.

We also wish to precise that we did use Factor analysis to compute our score. Both the Pearson correlation coefficient and the Spearman correlation score were extremely high with the score obtained with the PCA (0.9963 and 0.9972 respectively). We chose to keep the PCA score as the reading of the coefficient of each item is more straight-forward, but the ranking of respondents is not affected at all by the method adopted.

We hope these new elements have convinced the reviewer of the solidity of our Satisfaction score.

b) certain dimensions of determinants are not comprehensively captures such as environmental and experience of health seeking only has 2 and 3 variables, respectively.

We acknowledge that we could not capture all aspects of the healthcare experience, but we rely both on literature and on qualitative work in other components of the global project and on pilot phasis to identify aspects that were the most crucial in patients experience, and in which we would be able to detect some variability between healthcare seeking experiences.

6. PLOS authors have the option to publish the peer review history of their article ([what does this mean?](https://track.editorialmanager.com/CL0/https:%2F%2Fjournals.plos.org%2Fplosone%2Fs%2Feditorial-and-peer-review-process%23loc-peer-review-history/1/010f01973a942a42-5ce9b14c-de27-40cc-9767-48b85797a8f3-000000/f34oUVo69gIvnpuay7Do5h79U4a_ZMmEMWcL8gHJBis=214)). If published, this will include your full peer review and any attached files.


If you choose “no”, your identity will remain anonymous but your review may still be made public.


**Do you want your identity to be public for this peer review?** For information about this choice, including consent withdrawal, please see our [Privacy Policy](https://track.editorialmanager.com/CL0/https:%2F%2Fwww.plos.org%2Fprivacy-policy/1/010f01973a942a42-5ce9b14c-de27-40cc-9767-48b85797a8f3-000000/9CM-Qb4yIPRrE38suOe4AWKpSDP1beybY1XNVycSfJg=214).

Reviewer #1: **Yes:** SYED FARHAN ALI TIRMIZI

Reviewer #2: No

[NOTE: If reviewer comments were submitted as an attachment file, they will be attached to this email and accessible via the submission site. Please log into your account, locate the manuscript record, and check for the action link "View Attachments". If this link does not appear, there are no attachment files.]

While revising your submission, please upload your figure files to the Preflight Analysis and Conversion Engine (PACE) digital diagnostic tool, [https://pacev2.apexcovantage.com/](https://track.editorialmanager.com/CL0/https:%2F%2Fpacev2.apexcovantage.com%2F/1/010f01973a942a42-5ce9b14c-de27-40cc-9767-48b85797a8f3-000000/u_WB-tYSbLBah501uOVPlfsC6FUjvjiIRZGoB2QKwRA=214). PACE helps ensure that figures meet PLOS requirements. To use PACE, you must first register as a user. Registration is free. Then, login and navigate to the UPLOAD tab, where you will find detailed instructions on how to use the tool. If you encounter any issues or have any questions when using PACE, please email PLOS at [figures@plos.org](mailto:figures@plos.org). Please note that Supporting Information files do not need this step.
