# Supplementary material for: Examining individual, health service, and experience of care determinants of patients’ satisfaction - A cross-sectional facility-based study in KP, Pakistan
Source: PLoS One. 2026 May 27;21(5):e0346788. doi: 10.1371/journal.pone.0346788 (PMC13215502; doi:10.1371/journal.pone.0346788)
Supplement: Attachment — Submitted filename: rebuttal letter.docx [file pone.0346788.s007.docx]

PONE-D-25-05636R1

Examining individual, health service, and experience of care determinants of patients’ satisfaction - a cross-sectional facility-based study in KP, Pakistan

PLOS One

Dear Dr. Petitfour, 

We thank the reviewer for their careful reading of our manuscript, and provide answers to the remaining comments one by one.

6. Review Comments to the Author

Please use the space provided to explain your answers to the questions above. You may also include additional comments for the author, including concerns about dual publication, research ethics, or publication ethics. (Please upload your review as an attachment if it exceeds 20,000 characters)

**Reviewer #1: General Assessment
The manuscript tackles a very relevant, important topic. The data is unique, coming from several districts, and encompasses public, as well as private, facilities. The methodological toolkit is, overall, appropriate, and the manuscript is well-explained. However, there appear several aspects, mainly related to sampling and sample size, that need clarification, concerning the overall goal of the study, with regard to intrinsic coherence. These points do not make the manuscript incorrect, per se, but need correction.**

**MAJOR COMMENTS**

**1. Absence of Sample Size Justification: The manuscript does not provide a sample size calculation or justification, either at the facility or patient level.
Suggested action: Explicitly state whether the sample size was determined by feasibility/programmatic constraints, or a formal power calculation was conducted. If no formal calculation was done, authors should justify why the achieved sample (n=666) is sufficient for multivariable modelling, and acknowledge this as a limitation.**

We thank you for your comment, and your careful reading of the method section. We worked on the Study setting and the Sampling and interview setting subsections to make our sampling clearer and more explicit. Indeed, we did not rely on formal sample size calculations.

We present at the end of the 5 major comments the former and new version of these subsections.

**2. Unclear Primary Sampling Unit (Facility vs. Patient): The manuscript does not clearly define the sampling unit, and the description alternates between facilities as the unit of selection, and patients as the unit of analysis. This ambiguity affects interpretation of representativeness, variance estimation, and external validity.
Suggested action: Clearly state the primary sampling unit (PSU), the secondary sampling unit, and how this informed the analytical approach.**

We took your recommendation and explained in the text the primary sampling unit (the facility) and the secondary sampling unit (the patient).

We present at the end of the 5 major comments the former and new version of these subsections.

**3. Sampling Strategy: The described approach resembles a two-stage cluster sampling design, but this is never explicitly stated, nor analytically accounted for beyond clustering SEs.
Suggested action: Authors should explicitly describe the design as facility-based cluster sampling, acknowledge that patient selection was non-random, and discuss implications for generalizability.**

We explained in the text the two-stage clustering sampling strategy, and added a sentence in the limitations. Selection of patient within facilities was indeed not random, because the number of patients per day was not sufficient in many facilities to implement any consistent and random selection strategy.

We present at the end of the 5 major comments the former and new version of these subsections.

**4. Inconsistency ‘8 Interviews per Provider’ vs ‘per Facility’: There is inconsistency in the Methods section. In some places, the target is described as 8 interviews per provider; elsewhere, it is described as 8 interviews per facility. This is confusing, particularly given that facilities may include multiple providers.
Suggested action: Standardize terminology throughout the manuscript. Clarify whether the target was per facility-day, or per individual provider within facilities.**

We amended the terminology where it needed to be, and checked that it is consistent throughout the text.

**5. Shift in Study Objective (Satisfaction Determinants vs. Health Insurance)
The stated objective focuses on “…individual characteristics; health facility infrastructure; and patients’ experience with health service delivery.” However, health insurance (Sehat Plus Card) appears prominently in the analysis and discussion. The framing increasingly suggests insurance as a key exposure, despite outpatient services not being covered. This creates conceptual drift between stated objectives, analytical emphasis, and interpretation of findings.
Suggested action: Either explicitly include insurance/social protection as a determinant in the stated objectives, or reframe insurance as an exploratory or secondary exposure.**

We thank you for this comment which helped us improve the clarity of the manuscript. Indeed, this specific study is embedded in a larger implementation research project, which addresses the roll-out of a health insurance project. The four study districts were chosen for the purpose of this project and the focus on primary outpatient services was also derived from the research objective of this project.

On the contrary, the sampling of the facilities and of patients within facilities were done for the purpose of this specific study.

We initially thought bringing information about the broader project would be helpful, but in light of your comments, we think it creates more confusion, and removed precise reference to the project and its content.

**Former version**

Study setting

We collected data in four districts in the Khyber Pakhtunkhwa province of Pakistan: Chitral, Kohat, Malakand and Mardan. These districts were selected in agreement with the local health authorities as the target of an upcoming health insurance program targeting primary healthcare services. No health insurance targeting primary care was yet active at the time of data collection. The healthcare delivery system in Khyber Pakhtunkhwa relies on both public and private facilities (see Figure 1).

Public sector healthcare delivery follows a pyramidal structure: the bottom tier consists of Basic Health Units (BHU), which commonly include two to five medical practitioners that provide primary outpatient care for a modest fee of 10 PKR. The second tier consists of Rural Health Centers (RHCs), which can vary in both their size and their range of primary and some specialty services, as well as inpatient care. The top tiers include Tehsil Head Quarter (THQs) and District Head Quarter (DHQs) hospitals, which offer both inpatient and outpatient care for a range of specialty services. The private sector consists of individual or group-based general practitioners (GP) or specialists providing facility- or office-based care. Group-based private providers usually consist of two or more practitioners offering services at facilities managed by a single private authority. In both private and public sector facilities direct user charges are the predominant modality, although these charges are highly subsidized in the latter.

Sampling and interview setting

We collected data from September to November 2022 and from March to April 2023 from a sample of 60 public and 62 private healthcare facilities providing primary level outpatient services. The public sample included a census of all DHQ and THQ hospitals, all RHCs, and 25 BHUs randomly selected from a total of 98 BHUs in the study area. The private sample included 62 facilities randomly selected from a list of 245 private facility provided to us by the KP Health Department. The 62 private facilities included 37 GPs (27 individual and 10 group-based practices), 11 clinical specialists (six individual, five group-based), and seven group-based outpatient service practices linked to an inpatient care facility.

The sample size was constrained by the overall number of facilities providing services in the four districts and by the objective of the overall study hosting the analysis presented in this paper. The overall objective is to monitor the implementation of a health insurance scheme in the study area. Hence, our sample included a representative sample of all healthcare providers, public and private, providing primary care services across the four districts. We included all DHQs, THQs, and RHQs (as people also seek primary outpatient services at this level) - identified as hospitals - and a random sample stratified by district of all BHUs to ensure district representation. Similarly, we included a random sample of all private providers stratified by district, not by facility type due to absence of information. Health facility lists, setting the basic sampling frame, were handed to the research team by the local health authorities.

At each sampled primary healthcare facility, we conducted exit interviews with patients exiting the facility premises after having attended outpatient care services. Our teams were stationed at each facility for one day. The enumerators introduced themselves, and the study to the patients, and asked them for their consent to participate. They also asked a few questions to check their eligibility. At each facility, on a given day, we included any adult patient exiting general outpatient services who consented to take part to the study, irrespective of their condition. Children or disabled patients that could not respond to the questionnaire on their own were excluded. After consenting to the interview, enumerators sat in a quiet and isolated location in or around the facility with consenting patients and administered the interview, lasting about 20 minutes. Enumerators had a target of interviewing eight patients per provider. We sat a target of reaching a minimum of eight interviews per facility, but enumerators could interview more in larger facilities with greater patient attendance.

**New version**

Study setting

We collected data in four districts in the Khyber Pakhtunkhwa province of Pakistan: Chitral, Kohat, Malakand and Mardan. These districts were selected in agreement with the local health authorities as the target of an upcoming implementation project targeting primary healthcare services. The present study is embedded in the baseline survey of this project, so that no project activity had started at the time of data collection.

The healthcare delivery system in Khyber Pakhtunkhwa relies on both public and private facilities (see Figure 1). Public sector healthcare delivery follows a pyramidal structure: the bottom tier consists of Basic Health Units (BHU), which commonly include two to five medical practitioners that provide primary outpatient care for a modest fee of 10 PKR. The second tier consists of Rural Health Centers (RHCs), which can vary in both their size and their range of primary and some specialty services, as well as inpatient care. The top tiers include Tehsil Head Quarter (THQs) and District Head Quarter (DHQs) hospitals, which offer both inpatient and outpatient care for a range of specialty services. The private sector consists of individual or group-based general practitioners (GP) or specialists providing facility- or office-based care. Group-based private providers usually consist of two or more practitioners offering services at facilities managed by a single private authority. In both private and public sector facilities direct user charges are the predominant modality, although these charges are highly subsidized in the latter.

Sampling and interview setting

We used a two-stage cluster sampling strategy. We did not rely on sample size calculations, but rather aim at having enough patients in each type of facilities to identify individual and facility- level phenomenon. First, the number of facilities (our primary sampling unit) was defined to reflect the diversity of facilities offering primary outpatient services and remain feasible for field teams. The public sample of 60 facilities included a census of all DHQ and THQ hospitals, all RHCs, and 25 BHUs randomly selected from a total of 98 BHUs in the study area. We implemented a stratified selection of BHUs at the district level to ensure the district representation. The private sample included 62 facilities randomly selected from a list of 245 private facility provided to us by the KP Health Department. The 62 private facilities included 37 GPs (27 individual and 10 group-based practices), 11 clinical specialists (six individual, five group-based), and seven group-based outpatient service practices linked to an inpatient care facility. We implemented a stratified selection at the district level to ensure the district representation.

Second, we defined the number of patients (secondary sampling unit) to interview per sampled facility, aiming at capturing of individual situations and experiences, while remaining feasible within the time the surveyors could spend in each facility. We sat a minimum target of eight interviews per facility, but enumerators could interview more patients in larger facilities with greater patient attendance. We conducted exit interviews with patients exiting the facility premises after having attended outpatient care services. Our teams were stationed at each facility for one day. At each facility, on a given day, we included any adult patient exiting general outpatient services who consented to take part in the study, irrespective of their condition. The enumerators introduced themselves, and the study to the patients, and asked them for their consent to participate. They also asked a few questions to check their eligibility. Children or disabled patients that could not respond to the questionnaire on their own were excluded. Enumerators sat in a quiet and isolated location in or around the facility with consenting patients and administered the interview, lasting about 20 minutes. We collected data from September to November 2022 and from March to April 2023

**MINOR COMMENTS
Discussion
• Interpret the insurance finding more cautiously, emphasizing that causality cannot be inferred.**

We amended the text in this particular section, talking about association only between satisfaction and insurance
**Language & Style
• Minor grammatical edits needed (e.g., tense consistency, phrasing such as “abidance” vs. “adherence”).
• Some sentences are overly long and would benefit from simplification.**

We read the manuscript carefully and amended it where need.

7. PLOS authors have the option to publish the peer review history of their article ([what does this mean?](https://track.editorialmanager.com/CL0/https:%2F%2Fjournals.plos.org%2Fplosone%2Fs%2Feditorial-and-peer-review-process%23loc-peer-review-history/1/010f019b72b09469-84eb7149-a0de-4ebe-acb8-c60f3a3f58f3-000000/SccvuTVDmHWzJeX5h7DGbHJ55nxxHz4RqUwLnicrYAI=244)). If published, this will include your full peer review and any attached files.

If you choose “no”, your identity will remain anonymous but your review may still be made public.

**Do you want your identity to be public for this peer review?** For information about this choice, including consent withdrawal, please see our [Privacy Policy](https://track.editorialmanager.com/CL0/https:%2F%2Fwww.plos.org%2Fprivacy-policy/1/010f019b72b09469-84eb7149-a0de-4ebe-acb8-c60f3a3f58f3-000000/Hx31pV31VDrKjzUYayAOrpu7MhDFmynHXJ-Ry43JnU0=244).

Reviewer #1: **Yes:** SYED FARHAN ALI TIRMIZI
